# Acute kidney injury after living‐related liver transplantation in infants with biliary atresia: A retrospective study

**DOI:** 10.1002/pdi3.37

**Published:** 2023-10-30

**Authors:** Wei Liu, Xiaoke Dai, Hongxia Guo, Chengyan You, Min Du, Qiu Li

**Affiliations:** ^1^ Department of Anesthesiology Children's Hospital of Chongqing Medical University Chongqing China; ^2^ Ministry of Education Key Laboratory of Child Development and Disorders Chongqing Key Laboratory of Pediatrics Chongqing China; ^3^ National Clinical Research Center for Child Health and Disorders Chongqing China; ^4^ Department of Hepatobiliary Surgery Children's Hospital of Chongqing Medical University Chongqing China; ^5^ China International Science and Technology Cooperation Base of Child Development and Critical Disorders Chongqing China

**Keywords:** acute kidney injury, biliary atresia, infants, liver transplantation, pediatric

## Abstract

Although acute kidney injury (AKI) is a frequent postoperative complication after liver transplantation, renal function following living‐related liver transplantation (LRLT) in infants remains understudied. The aim of this study was to identify the prevalence of AKI and the impact of AKI on outcomes in infant recipients with biliary atresia. We retrospectively reviewed infants who underwent LRLT surgery between January 2018 and January 2021. The primary outcome was the risk of postoperative AKI occurrence in the first 7 postoperative days. A multivariate regression analysis model was used to investigate risk factors for AKI, and the incidence and impacts of AKI on outcomes were analyzed. A total of 98 patients were included in the analysis. AKI occurred in 59 patients (60.2%) within 7 days after surgery. Logistic regression analysis revealed that preoperative transfusion (*p* < 0.05) and lower serum creatinine (SCr) (*p* < 0.05) were independent risk factors for AKI. The incidence of serious complications was significantly higher in the AKI group than in the non‐AKI group (*p* < 0.05). The postoperative mechanical ventilation time (*p* < 0.05) and hospital stay (*p* = 0.019) were significantly longer in the AKI group. There was no evidence of chronic kidney disease (CKD) in any surviving infants within 1 year after surgery. In conclusion, AKI is common in infant LRLT (60.2%), and preoperative transfusion and lower SCr levels were independently associated with AKI. AKI may be associated with the incidence of serious complications, prolonged use of ventilators, and hospital stays. No CKD occurred within 1 year.

## Graphical abstract

Acute kidney injury (AKI) is common in infant LRLT, and preoperative transfusion and lower serum creatinine levels were independently associated with AKI. AKI may be related to prolonged use of ventilators and hospital stays, and no chronic kidney disease occurred within 1 year.

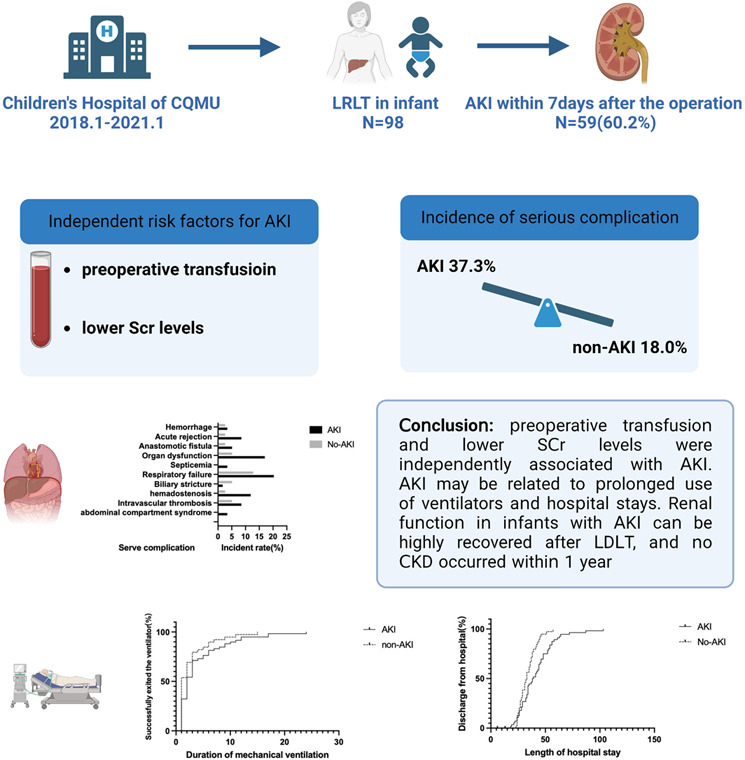

## 1 INTRODUCTION

Pediatric living‐related liver transplantation (LRLT) is an effective method for the treatment of end‐stage liver disease. LRLT is a solution for the serious shortage of liver sources and reduces the mortality of children waiting for transplantation.^1^ Among all children with end‐stage liver disease, congenital biliary atresia (BA) has become the most common indication for pediatric liver transplantation due to its high incidence, accounting for approximately 60%–70% of all adaptive cases, with more than 90% of those cases diagnosed in infancy.^2^ The number of pediatric liver transplantations in mainland China has increased significantly, and infants have gradually become the main recipient group of pediatric liver transplantations with approximately 1000 cases per year.^3^ Economic development, advances in medical technology, and the accumulation of experience have increased the willingness of Chinese families to accept LRLT, and at our medical center, approximately 80% of recipients are infants younger than 1 year old who receive part of their father's or mother's liver.

Acute kidney injury (AKI) is a well‐known complication in liver transplant recipients with a frequency ranging between 17% and 90%.^4^, ^5^ In studies involving adults and children, perioperative data were analyzed to identify independent risk factors for posttransplant AKI.^6^, ^7^ AKI is also associated with worse outcomes, including longer intensive care unit (ICU) and hospital stays, decreased short‐term and long‐term graft survival, increased postoperative infection rates, and the development of chronic kidney disease (CKD).^8^, ^9^ Only a few studies have focused on the risk of AKI following pediatric liver transplantation, which has been associated with prolonged hospital and ICU stays and increased mortality.^10^, ^11^, ^12^

However, there is little research reporting the incidence and outcomes of AKI after LRLT in infants. Most of the data from previous studies came from a wide age range of recipients (0–18 years of age), and the different types of liver disease were analyzed together. Therefore, we performed a retrospective study to determine the risk of AKI occurrence after LRLT in infants with BA. In addition, we investigated renal function recovery and outcomes within 1 year.

## 2 METHODS

### 2.1 Design

This single‐center retrospective study was conducted at the Children's Hospital of Chongqing Medical University (CHCMU) in China. The current retrospective study was approved by the ethics committee of CHCMU (approval number: 2020‐284). Patients were identified using electronic healthcare data, which included their medical and medication history, procedure records, and laboratory results that were maintained in the study setting. All data were collected and analyzed by specialized data researchers on our team. All data were managed by project leaders and specialized data researchers to safeguard the integrity of the data and the privacy of patients.

### 2.2 Study population and data collection

All infants (<1 year) who were diagnosed with BA and received LRLT at our institution from January 2018 to January 2021 were included. All children received living‐related left hemi‐liver grafts. Exclusion criteria included combined liver‐kidney transplantation, previous AKI, chronic renal failure, multiple liver transplants, and lack of sufficient laboratory or clinical data.

We collected preoperative, intraoperative, and postoperative clinical data and laboratory test results. Preoperative data included patient demographics, Pediatric Model for End‐Stage Liver Disease (PMELD) scores, blood transfusion, and baseline laboratory test variables. Intraoperative data included operation time, warm and cold ischemia time, anhepatic phase, graft‐recipient weight ratio, blood products and fluid transfusion, blood loss, urine volume, and occurrence of reperfusion syndrome.

### 2.3 Intraoperative management protocol

Standardized anesthesia management protocols are important to minimize the influence of intraoperative factors on postoperative AKI. The choice of anesthetic agents, fluid therapy, hemodynamic stability, internal environment stability, and blood conservation strategies may affect the outcomes. At our institution, anesthetics for all liver transplants were administered by the same team of anesthesiologists, and standardized anesthesia and intraoperative management strategies were used. General anesthesia was induced with 0.05 mg/kg midazolam, 2 mg/kg propofol, 1 μg/kg sufentanil, and 0.1 mg/kg homeopathic atracuride. During the surgery, anesthesia was maintained with propofol (4 mg/kg/h) and/or sevoflurane combined with remifentanil (0.2 μg/kg/min). Autologous blood recovery devices were used in all children, and the internal environment and composition of blood transfusion were adjusted according to the results of arterial blood gas analysis. Norepinephrine was the first‐line vasopressor of choice; if norepinephrine was ineffective, then dopamine, epinephrine, and pituitrin were considered. The posttransplant immunosuppression regimen was determined at the discretion of the hepatobiliary surgery team.

### 2.4 Study outcomes and definitions

The primary outcome was the risk of postoperative AKI occurrence according to the Kidney Disease: Improving Global Outcomes (KDIGO) criteria.^13^ It is staged as follows: Stage 1: Increase in serum creatinine (SCr) to 1.5–1.9 times baseline, increase in SCr by ≥0.3 mg/dL, or reduction in urine output to <0.5 mL/kg/h for 6–12 h. Stage 2: Increase in SCr to 2.0–2.9 times baseline or reduction in urine output to <0.5 mL/kg/h for ≥12 h. Stage 3: Increase in SCr to 3.0 times baseline, increase in SCr to ≥4.0 mg/dL, reduction in urine output to <0.3 mL/kg/h for ≥24 h, anuria for ≥12 h, initiation of kidney replacement therapy, or in patients <18 years, decrease in estimated glomerular filtration rate to <35 mL/min/1.73 m^2^.

Postoperative AKI stages were based on the SCr concentration measured daily and urine output measured every 2 h in the first 7 postoperative days. Patients were divided into an AKI group and a non‐AKI group according to these criteria. Secondary outcomes included the incidence of mild and severe complications, duration of mechanical ventilation, length of ICU and hospital stays, incidence of mortality, and renal function within 1 year after surgery. Pneumonia, atelectasis, pleural effusion, abdominal effusion, chylous fistula, and infection that were nonlife‐threatening were defined as mild complications. Abdominal compartment syndrome, intravascular thrombosis, hemadostenosis, biliary stricture, respiratory failure, septicemia, organ dysfunction, anastomotic fistula, acute rejection, and hemorrhage were defined as serious complications. We assessed kidney function in surviving infants every month for 1 year after surgery.

### 2.5 Statistical analysis

Continuous measures are reported as the mean ± standard deviation or median and interquartile range. We performed a univariate analysis for each potential risk factor, including preoperative demographic characteristics, laboratory tests, and intraoperative data, to determine whether there was an association between variables and the development of AKI. For continuous variables, univariate analysis was performed with *T* tests or Wilcoxon rank‐sum tests; for categorical parameters, chi‐square tests or Fisher exact tests for categorical measures were performed. Based on the results of the univariate variable analysis, we took the variables with *p* < 0.05 as the potential predictors that were significantly associated with AKI and further analyzed the above potential predictors through the multivariate logistic regression analysis model to determine the correlation between them and AKI occurrence.

All tests were two‐tailed, and a *p* value of <0.05 was considered statistically significant. The results are reported as odds ratios (ORs) with 95% confidence intervals. Data were analyzed using SPSS version 20.0 (IBM Corporation).

## 3 RESULTS

**FIGURE 1:**
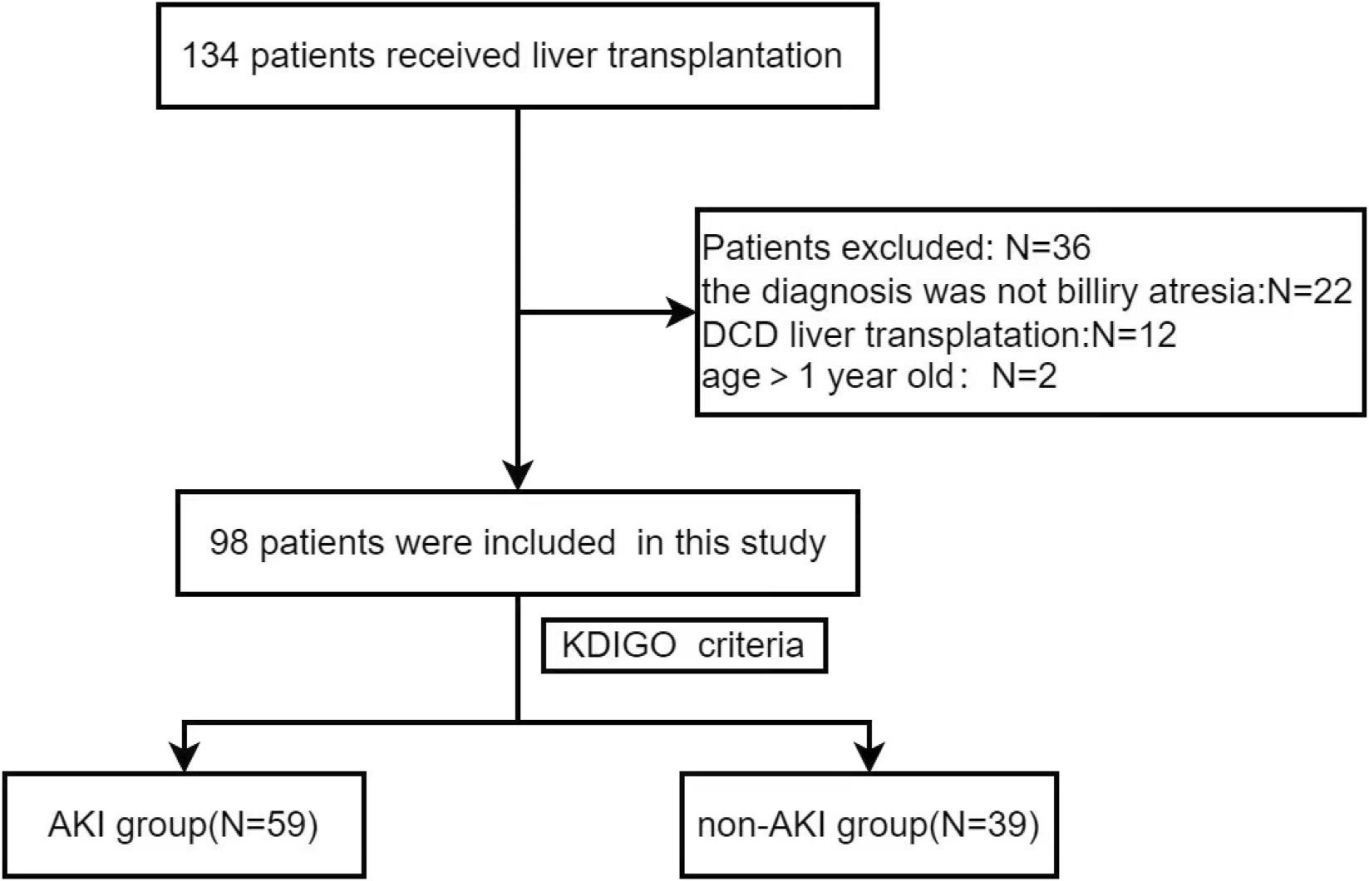
Flowchart of patient selection. AKI, acute kidney injury; DCD, donation after circulatory death; KDIGO, Kidney Disease: Improving Global Outcomes.

A total of 134 LRLTs were performed. Thirty‐six patients were excluded: 22 patients were not diagnosed with BA, 12 patients received donation after circulatory death transplantation, and 2 patients were out of the infant age range. A total of 98 patients were included in the final analysis (Figure 1).

### 3.1 Risk of AKI occurrence after LRLT

Among these patients, 59 (60.2%) developed AKI within 7 days after the operation. There were 29 patients (29.6%) with stage 1 AKI, 18 patients (18.4%) with stage 2 AKI, and 12 patients (12.2%) with stage 3 AKI.

### 3.2 Risk factors for AKI after LRLT

The preoperative characteristics and intraoperative data of infants with AKI versus non‐AKI are shown in Tables 1, 2, 3, 4, 5. Univariate results revealed that preoperative transfusion (*p* = 0.023), lower albumin levels (*p* = 0.032), and lower SCr levels (*p* = 0.001) were associated with the development of AKI. For intraoperative data, prolonged operation time (*p* = 0.039), anhepatic stage time (*p* = 0.029), and cold ischemia time (*p* = 0.027) were associated with the development of AKI. According to multivariable logistic regression analysis, preoperative transfusion (*p* = 0.047, OR = 0.318, 95% CI: 0.103–0.984) and lower SCr preoperatively (*p* = 0.001, OR = 0.772, 95% CI: 0.660–0.903) were independently associated with AKI (Table 6).

**TABLE 1:** Patient characteristics.

Variable	All patients (*n* = 98)	AKI (*n* = 59)	Non‐AKI (*n* = 39)	*p* value
Age (Mon)	5.30 (4.73, 6.23)	5.33 (4.73, 6.23)	5.17 (4.73, 6.23)	0.850
Sex, male (*n* [%])	54 (56.1)	36 (61.0)	19 (48.7)	0.299
Weight (kg)	6.63 ± 1.11	6.78 ± 1.17	6.41 ± 0.98	0.100
PELD score	16.7 (13.6, 21.6)	17.6 (14.5, 22.1)	15.1 (12.2, 20.4)	0.139
GRBW	0.03 (0.03, 0.04)	0.03 (0.03, 0.04)	0.03 (0.03, 0.04)	0.860
Preoperative blood transfusion, (*n* [%])	27 (27.6)	21 (35.6)	6 (15.4)	0.023

*Note*: Values are presented as the mean ± standard deviation, median (IQR), or number (%).

Abbreviations: AKI, acute kidney injury; GRBW, graft to recipient body weight (hepatology); IQR, interquartile range; PELD score, pediatric end‐stage liver disease score.

**TABLE 2:** Liver function laboratory data.

Variable	All patients (*n* = 98)	AKI (*n* = 59)	Non‐AKI (*n* = 39)	*p* value
TB (μmol/L)	292 (222, 368)	301 (241, 381)	274 (204, 355)	0.559
TP (g/L)	62.4 ± 9.0	61.3 ± 9.2	64.1 ± 8.6	0.141
ALB (g/L)	35.6 (30.5, 38.8)	34.1 (30.1, 37.1)	37.5 (31.3, 41.0)	0.032
GLOB (g/L)	24.7 (20.3, 34.1)	24.7 (20.3, 35.0)	25.6 (20.8, 32.6)	1.000
ALT (U/L)	225 (149, 319)	215 (143, 294)	283 (169, 350)	0.058
AST (U/L)	399 (281, 611)	382 (281, 494)	435 (274, 830)	0.181
AST/ALT	1.84 (1.52, 2.48)	1.90 (1.53, 2.59)	1.82 (1.50, 2.30)	0.814
ALP (U/L)	676 (508, 914)	678 (500, 951)	621 (511, 807)	0.658
GGT (U/L)	318 (97, 649)	363 (153, 671)	284 (76, 641)	0.204
LDH (U/L)	353 (286, 410)	360 (303, 413)	328 (285, 409)	0.566
CHE (U/L)	3162 (2455, 4077)	3031 (2307, 3735)	3438 (2585, 4794)	0.219

*Note*: Values are presented as the mean ± standard deviation, median (IQR).

Abbreviations: AKI, acute kidney injury; ALB, albumin; ALP, alkaline phosphatase; ALT, alanine transaminase; AST, aspartate aminotransferase; CHE, cholinesterase; GGT, gamma‐glutamyl transpeptidase; GLOB, globulin; IQR, interquartile range; LDH, lactate dehydrogenase; TB, total bilirubin; TP, total protein.

**TABLE 3:** Renal function and electrolytes.

Variable	All patients (*n* = 98)	AKI (*n* = 59)	Non‐AKI (*n* = 39)	*p* value
BUN (mmol/L)	2.34 (1.90, 3.03)	2.20 (1.80, 2.75)	2.50 (2.00, 3.10)	0.086
Scr (μmol/L)	14.5 (13.0, 17.0)	14.0 (12.0, 16.0)	16.5 (14.0, 19.0)	0.001
UA (μmol/L)	108 (82, 145)	103 (80, 140)	120 (89, 158)	0.154
K^+^ (mmol/L)	4.75 ± 0.48	4.81 ± 0.45	4.67 ± 0.53	0.183
Na^+^ (mmol/L)	137 ± 2.8	136 ± 2.9	137 ± 2.8	0.067
Cl^+^ (mmol/L)	104.0 ± 3.1	103.6 ± 2.9	104.7 ± 3.2	0.076

*Note*: Values are presented as the mean ± standard deviation, median (IQR).

Abbreviations: AKI, acute kidney injury; BUN, blood urea nitrogen; IQR, Interquartile range; Scr, serum creatinine; UA, uric acid.

**TABLE 4:** Routine blood examination and coagulation function.

Variable	All patients (*n* = 98)	AKI (*n* = 59)	Non‐AKI (*n* = 39)	*p* value
HCT (%)	30.3 (26.7, 32.6)	30.2 (26.4, 32.6)	30.4 (28.2, 32.6)	0.315
WBC (10^9^/L)	13.9 (10.5, 18.6)	13.1 (10.5, 19.0)	14.42 (10.2, 16.9)	0.865
Plt (10^9^/L)	239 (177, 325)	256 (185, 333)	231 (162, 304)	0.157
Hb (g/L)	99.0 ± 14.0	96.0 ± 13.5	102 ± 14.4	0.071
N%	0.38 ± 0.15	0.39 ± 0.14	0.38 ± 0.16	0.826
L%	0.56 (0.45, 0.67)	0.55 (0.46, 0.65)	0.58 (0.43, 0.69)	0.599
MON%	0.04 (0.03, 0.04)	0.04 (0.03, 0.04)	0.04 (0.03, 0.04)	0.475
EO%	0.02 (0.01, 0.03)	0.02 (0.01, 0.02)	0.02 (0.01, 0.03)	0.712
PT (s)	13.7 (12.4, 17.7)	14.3 (12.5, 17.9)	13.0 (12.2, 17.6)	0.325
APTT (s)	33.7 (29.9, 38.9)	33.9 (30.2, 38.6)	33.3 (29.9, 39.6)	0.760
TT (s)	20.1 (18.5, 22.4)	20.0 (20.0, 18.4)	21.1 (18.6, 22.7)	0.627
PTR	1.19 (1.07, 1.54)	1.23 (1.08, 1.57)	1.13 (1.06, 1.51)	0.318
Fib (g/L)	1.77 ± 0.73	1.82 ± 0.75	1.69 ± 0.71	0.397
D‐Dim (mg/L)	1.62 (0.81, 4.70)	1.76 (0.94, 4.75)	1.25 (0.57, 4.27)	0.204

*Note*: Values are presented as the mean ± standard deviation, median (IQR).

Abbreviations: AKI, acute kidney injury; APTT, activated partial thromboplastin time; D‐Dim, D dimer; EO%, eosinophils percentage; Fib, fibrinogen; Hb, hemoglobin; HCT, hematocrit; IQR, interquartile range; L%, lymphocyte percentage; MON%, monocyte percentage; N%, neutrophil percentage; Plt, platelets; PT, prothrombin time; PTR, prothrombin time ratio; TT, thrombin time; WBC, white blood cell.

**TABLE 5:** Intraoperative factors.

Variable	All patients (*n* = 98)	AKI (*n* = 59)	Non‐AKI (*n* = 39)	*p* value
Operation time (min)	465 (420, 516)	477 (430, 540)	445 (415, 490)	0.039
Cold ischemia time (min)	122 (92, 148)	136 (99, 153)	109 (87, 134)	0.027
Warm ischemia time (min)	2 (1, 2)	2 (1, 2)	2 (1, 2)	0.591
Anhepatic phase (min)	56 (48, 64)	58 (49, 70)	53 (47, 60)	0.029
Blood loss (mL/kg)	66.7 (37.5, 138.0)	67.8 (42.4, 150.0)	63.6 (33.3, 107.7)	0.147
Total fluid intake (mL)	950 (732, 1276)	1026 (775, 1315)	815 (705, 1190)	0.152
RBC transfusion (u)	2 (1.5, 2.5)	2.0 (1.5, 2.5)	1.5 (1.0, 2.0)	0.064
FFP transfusion (u)	100 (0, 200)	150 (0, 200)	100 (0, 200)	0.199
20% albumin (g)	32.5 (27.5, 40)	35.0 (27.5, 40.0)	30.0 (20.0, 40.0)	0.353
Cryoprecipitate (u)	0 (0, 2)	0 (0, 2)	0 (0, 2)	0.589
Urine volume (mL)	250 (154, 405)	225 (155, 400)	265 (150, 500)	0.490

*Note*: Values are presented as the mean ± standard deviation, median (IQR), or number (%).

Abbreviations: AKI, acute kidney injury; FFP, fresh frozen plasma; IQR, Interquartile range; RBC, red cell suspension.

**TABLE 6:** Logistic regression analysis of patients with and without AKI.

Variable	*p*	OR	95% CI
Preoperative SCr (μmol/L)	0.001	0.772	0.660–0.903
Preoperative blood transfusion	0.047	0.318	0.103–0.984
ALB (g/L)	0.743	1.006	0.969–1.045
Operation time (min)	0.185	1.004	0.998–1.011
Cold ischemia time (min)	0.418	1.007	0.990–1.026
Anhepatic phase (min)	0.294	1.028	0.976–1.083

*Note*: The ORs are given per increase by one unit and adjusted for all continuous variables shown in the table.

Abbreviations: AKI, acute kidney injury; ALB, albumin; CI, confidence interval; OR, odds ratio; SCr, serum creatinine.

### 3.3 Mortality and outcomes

The 3 patients who died in the hospital were all from the AKI group, and all were stage 3 patients. The in‐hospital mortality rate of stage 3 infants was 25%, which was significantly higher than that of stages 1 and 2 AKI patients (*p* = 0.007). After discharge from the hospital, there were 3 deaths in the AKI group (1 died of respiratory failure, 1 died of intestinal necrosis and 1 died of sepsis) and 2 deaths in the non‐AKI group (1 died of sepsis and another died of multiple organ dysfunction) within 1 year after surgery. The 1‐year survival rates of the non‐AKI and AKI groups were 94.8% and 89.8%, respectively. There were no significant differences in in‐hospital mortality or 1‐year mortality between the two groups.

**FIGURE 2:**
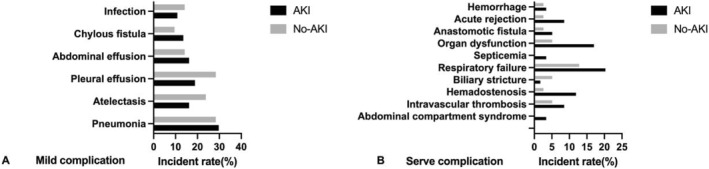
Incidence of mild (A) and severe complications (B) in both groups. AKI, acute kidney injury.

**FIGURE 3:**
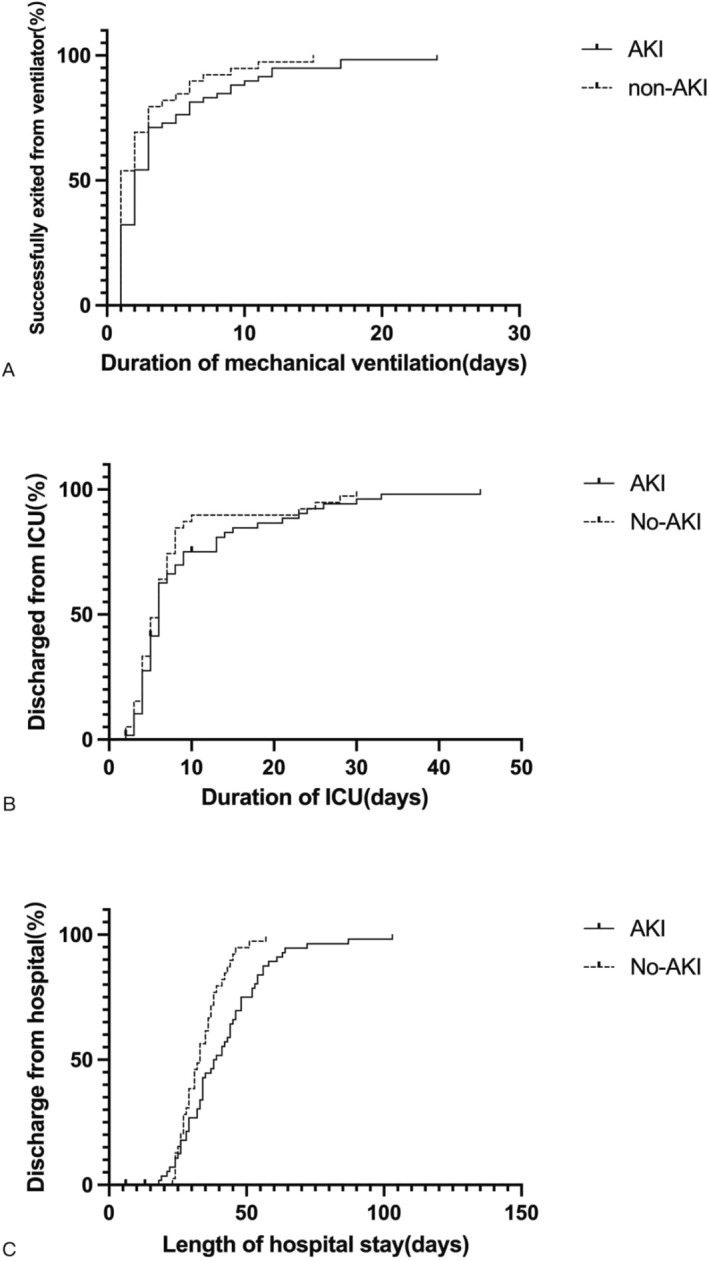
Duration of mechanical ventilation (A), duration of ICU stay (B), and length of hospital stay (C) in both groups. AKI, acute kidney injury; ICU, intensive care unit.

The incidence of serious complications was significantly higher in the AKI group than in the non‐AKI group (37.3% vs. 18.0%, *p* = 0.045). There was no significant difference in the incidence of mild complications (61.0% vs. 48.7%, *p* = 0.299). The incidence of various postoperative in‐hospital complications is shown in Figure 2. The durations of postoperative mechanical ventilation (2 [1,5] vs. 1 [1,3], *p* = 0.046) and hospital stay (38 [28,48] vs. 33 [27,38], *p* = 0.013) in the AKI group were significantly longer than those in the non‐AKI group, but there was no significant difference in length of ICU stay between the two groups (6 [4,9] vs. 6 [4,8], *p* = 0.485) (Figure 3).

Renal function was followed up every month in all discharged patients. There was no evidence of CKD in any of the surviving patients.

## 4 DISCUSSION

In this retrospective study of 98 infant LRLT recipients, we found that the prevalence of postoperative AKI according to the KDIGO criteria was 60.2%, with 29.6% in stage 1, 18.4% in stage 2%, and 12.2% in stage 3. The few studies that focus on AKI after pediatric liver transplantation show that the incidence is approximately 34.2%–57%.^9^, ^10^, ^11^, ^12^, ^13^, ^14^ Hamada et al reported that the risk of postoperative AKI in patients aged between 3 months and 18 years was 46.2%, including 21.8% with stage 1, 20.5% with stage 2%, and 3.8% with stage 3. Sahinturk et al. recently reported that the risk of AKI in patients <18 years was 34.2%, including 18% with stage 1, 10.2% with stage 2%, and 6% with stage 3. Although the overall occurrence of AKI was similar in existing studies, notably, the proportion of stage 3 AKI in our study was larger than that in previous studies.

Many studies in children and adults demonstrated that preoperative elevated total bilirubin level, activated partial thromboplastin time (APTT), PMELD score, intraoperative blood loss, prolonged cold ischemic time, duration of the anhepatic phase, red blood cell count, and fresh frozen plasma infusion are independent risk factors for AKI after liver transplantation.^6^, ^10^ The results of our study are different from those of adult liver transplantation studies, which suggest that preoperative elevated SCr is a risk factor of AKI.^15^ Low muscle content and poor nutritional status in infants will significantly reduce endogenous creatinine levels and thus affect plasma creatinine measurements.^16^ As renal perfusion gradually increases after birth, vascular resistance gradually decreases, and the glomerular filtration rate reaches adult levels around age 2.^17^, ^18^ In addition, infant renal tubules are immature and have a poor concentration capacity and a low capacity to reabsorb electrolytes and proteins.^19^ Few increases in plasma creatinine measurements can be a diagnosis of AKI, so even small changes in creatinine levels in infants should be taken seriously. Some children receiving LRLT may require preoperative blood transfusion due to the following factors: (1) the presence of moderate‐to‐severe anemia before surgery; (2) the presence of coagulation abnormalities with significantly prolonged APTT/PT before surgery; and (3) preoperative plasma exchange. Our study found that preoperative blood transfusion is an independent risk factor for AKI, which may be related to kidney injury caused by the immune response induced by blood transfusion.^20^ A longer operation time may induce postoperative AKI because of the complexity of liver transplantation surgery, especially for infants with delicate blood vessels, and biliary treatment may take a longer time. A longer operation time increases the potential for organ hypoperfusion, and the inflammatory response of tissue cells may cause damage to renal vessels and tissues.^21^, ^22^ The anhepatic stage after portal vein occlusion is the critical period of the surgical procedure. During this period, there is a sharp decrease in cardiac blood volume, congestion of the inferior vena cava system, tissue ischemia and hypoxia, increased anaerobic metabolism, and serious acidosis, all of which may aggravate kidney injury. Jochmans^23^ indicated that ischemia–reperfusion injury is an important factor in the occurrence of AKI after liver transplantation, and cold ischemia time was a common independent risk factor in previous studies.^24^ In this study, the univariate analysis showed that the three periods were different between the two groups, and the regression analysis showed that they were not independent risk factors. We postulate that the main reasons are as follows: (1) the disease course and similar anatomical conditions in all the patients; (2) minimal influence on hemodynamics after inferior vena cava occlusion in infants; and (3) continuous hypothermic perfusion of the liver immediately after leaving the donor significantly reduced donor liver injury. In our article, analysis by multivariate regression mostly included clinical tests and recorded values. In the absence of appropriate, acceptable clinical grading of these continuous variables, blindly grading data to simplify may miss some opportunities to discover new patterns. Therefore, based on the results of univariate analysis, this study further conducted multivariate logistic regression analysis for variables with *p* < 0.05.

Previous studies in adults^25^, ^26^, ^27^ and children^5^, ^7^, ^9^, ^11^ have shown that AKI is associated with increased morbidity and significantly longer hospital and ICU stays. In this study, no correlation was found between ICU stay and postoperative AKI, but the incidence of serious complications and mechanical ventilation time was significantly increased, which is consistent with previous research. Impaired kidney function may result in the retention of water and sodium, which may aggravate exudative lung lesions and hypoxemia, thereby prolonging ventilator use.

However, in our study, most AKI cases were self‐limiting without specific treatment. However, the occurrence of AKI can still significantly prolong the duration of mechanical ventilation and hospitalization. On the other hand, the absence of observed cases of CKD may be related to the fact that our follow‐up period was only 1 year. Several previous studies have demonstrated that AKI increases the risk of developing CKD and decreases graft and recipient survival,^5^, ^28^ thereby negatively affecting recipient prognosis and compromising clinical outcomes.^29^ Therefore, identification of risk factors for the development of AKI after liver transplantation and early intervention to prevent AKI becomes relevant to improve the outcome of this group of children after LRLT. Over the past years, several studies have shown a prevalence of CKD in pediatric liver transplantation ranging from 0% to 32%.^30^, ^31^ The major causes of renal dysfunction after liver transplantation include preexisting renal disease, perioperative renal injury, and nephrotoxicity of calcineurin inhibitors.^32^ This wide range is the result of different perioperative management strategies, anesthesia strategies, and immunosuppressive regimens across centers.^33^ Since kidney issues within 1 year after liver transplantation are often related to acute injury, the observation time in our study was 1 year, but no evidence indicating renal function injury was obtained in any of the patients with AKI. One of the possible reasons is that our team focuses primarily on protecting renal function during the operation, and early treatment measures, such as improving hemodynamics to maintain perfusion pressure, maintaining urine volume, correcting acid‒base imbalance and electrolyte disorder, increasing colloid osmotic pressure, and avoiding fluid overload, are adopted. Another reason may be the characteristics of renal capillaries and the strong renal repair ability of infants themselves, but the mechanism is not clear.

There are some limitations to our study. First, this is a single‐center study with a limited sample size, and our anesthesia management and perioperative medication programs do not represent the general situation. Second, we only used the KDIGO criteria for AKI, and the application of different criteria may lead to a different report of AKI prevalence and severity. Additionally, due to the small number of patients having incomplete medical and anesthetic records, some indicators were not analyzed as risk factors, leading to some potential variables being ignored. Finally, we only observed the recovery of renal function 1 year after surgery, which is a relatively short period for long‐term results. Because renal function may begin to deteriorate 2 or even 7–10 years after liver transplantation, long‐term outcomes need to be supported by more data obtained from longer follow‐ups.^34^ In our next study, we will further extend the follow‐up period to observe the recovery of long‐term renal function in children with LRLT. Additionally, we will further compare the perioperative management and postoperative AKI of LRLT in children with BA and non‐BA to better elucidate the perioperative anesthetic management of LRLT in different disease types.

## 5 CONCLUSIONS

AKI is common in infant LRLT (60.2%), and preoperative transfusion and lower SCr levels were independently associated with AKI. AKI may be associated with the incidence of serious complications and prolonged use of ventilators and hospital stays. Renal function in infants with AKI can be highly recovered after LRLT, and no CKD occurred within 1 year.

## AUTHOR CONTRIBUTIONS

Wei Liu and Min Du wrote the manuscript. Xiaoke Dai and Hongxia Guo helped conduct the study. Wei Liu and Chengyan You collected and analyzed the data. Min Du and Qiu Li initiated the idea, guided the article structure, and reviewed the final manuscript. All the authors have read and approved the final manuscript.

## CONFLICT OF INTEREST STATEMENT

The author(s) declare no potential conflicts of interest with respect to the research, authorship, and/or publication of this article. Qiu Liu is the Editor‐in‐Chief of Pediatric Discovery. To minimize bias, she was excluded from all editorial decision‐making related to the acceptance of this article for publication.

## ETHICS STATEMENT

The current retrospective study was approved by the ethics committee of CHCMU (approval number: 2020‐284).

## CONSENT TO PARTICIPATE

The requirement for obtaining informed consent was waived due to the retrospective study design.

## Data Availability

Research data are not shared.
